# Obstetric and psychosocial risk factors for Australian-born and non-Australian born women and associated pregnancy and birth outcomes: a population based cohort study

**DOI:** 10.1186/s12884-015-0681-2

**Published:** 2015-11-09

**Authors:** Hannah Grace Dahlen, Bryanne Barnett, Jane Kohlhoff, Maya Elizabeth Drum, Ana Maria Munoz, Charlene Thornton

**Affiliations:** School of Nursing and Midwifery, Western Sydney University, Locked Bag 1797, Penrith, NSW 2751 Australia; School of Psychiatry, Clinical Director, St John of God Raphael Centre, Medicine, University of New South Wales, 36-38 First Avenue, Blacktown, NSW 2148 Australia; St John of God Raphael Centre Blacktown, 36-38 First Ave, Blacktown, 2148 NSW Australia; Clinical Midwifery Consultant, Blacktown Hospital, Blacktown, Australia; Karitane, P.O. Box 241, Villawood, 2163 NSW Australia

## Abstract

**Background:**

One in four Australians is born overseas and 47 % are either born overseas or have a parent who was. Obstetric and psychosocial risk factors for these women may differ.

**Method:**

Data from one Sydney hospital (2012–2013) of all births recorded in the ObstetriX™ database were analysed (*n* = 3,092). Demographics, obstetric and psychosocial risk profile, obstetric interventions and complications and selected maternal and neonatal outcomes were examined for women born in Australia and overseas.

**Results:**

Women born in Australia were younger, more likely to be primiparous (28.6 v 27.5 %), be obese (32.0 % v 21.4 %), smoke (19.7 % v 3.0 %), have an epidural (26.2 % v 20.2 %) and were less likely to have gestational diabetes mellitus (GDM) (6.8 % v 13.7 % when compared to non-Australian born women. The highest rates of GDM, Gestational Hypertension (GH) and maternal anaemia were seen in women born in China, the Philippines and Pakistan respectively. Differences were also seen in psychosocial screening between Australian and non-Australian women with Australian-born women more likely to smoke and report a mental health disorder. There was an association between having an Edinburgh Postnatal Depression Scale (EPDS) ≥ 13 and other psychosocial issues, such as thoughts of self-harm, domestic violence, childhood abuse etc. These women were also less likely to breastfeed. Women with an EPDS ≥ 13 at booking compared to women with EPDS ≤12 had a higher chance of being diagnosed with GDM (AOR 1.85 95 % CI 1.14–3.0).

**Conclusions:**

There are significant differences in obstetric and psychosocial risk profiles and maternal and neonatal outcomes between Australian-born and non-Australian born women. In particular there appears to be an association between an EPDS of ≥13 and developing GDM, which warrants further investigation.

## Background

More than one in four Australians are born overseas (27 %), with 47 % either born overseas or having a parent who was born overseas [1, 2]. Over 300 separate ancestries were identified in the 2011 Australian Census. Nineteen percent of Australians speak another language than English and 2 % speak no English at all [2]. Around 82 % of the population born overseas live in capital cities such as Sydney compared to 66 % of all people in Australia [2]. While most of these migrants initially came from countries in North Western Europe (such as the UK), then Southern and Eastern Europe (such as Italy), this has now changed and migrants are increasingly arriving from China (6 %) and India (5.6 %). India (13 %) was the leading birthplace for migrants arriving in Australia between 2007–2011.

**Fig. 1:**
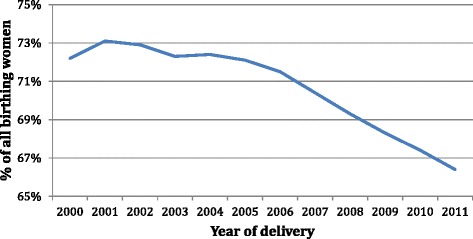
Country of birth Australia expressed as a % of all birthing women over time (NSW 2000-2011)

**Fig. 2:**
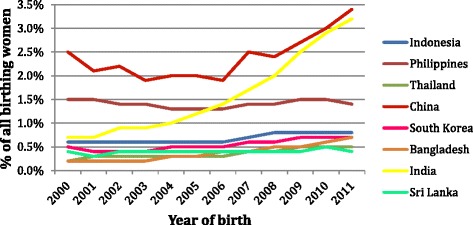
Country of birth expressed as a % of all birthing women over time (NSW2000-2011)

In New South Wales (NSW) between 2000 and 2011 there was a decline in women giving birth who were born in Australia from 72 % down to 66 % (Fig. 1) and a rise in the number of women giving birth who were born in China and India (Fig. 2) [3]. Some migrant groups, in particular women born in India, have very high rates of adverse antenatal events, obstetric intervention in birth and poorer neonatal outcomes compared to Australian-born women or women from other migrant groups, such as those born in Lebanon [3].

While many public antenatal clinics have introduced specific services for migrant women following the identification of increased risk factors and poorer outcomes, there is less awareness of the specific psychosocial support pregnant women from different migrant groups may need. Migrant women are more likely to have experienced previous trauma, loss of family members, social support and grief over loss of culture. With fewer family members and friends, feelings of social isolation and loneliness can be intense, placing them at greater risk of developing anxiety and depression. Lack of access to information and support, inadequate service provision, poor partner support, isolation and absent cultural practices increase the risk of developing perinatal mood disorders; especially where there is also a past history of anxiety and depression.

The Australian government places a strong emphasis on the management of mental health issues with particular focus on early intervention, social inclusion and recovery and service access, coordination and continuity of care. In Australia one in five women with a full-term infant has a current mental health condition or meets the DSM-V criteria for perinatal mental health problems in the first year following birth [4, 5]. Two thirds of women with depression or anxiety following the birth were symptomatic during the pregnancy with migrant women more likely to be affected with postnatal depression (PND) (24–42 % compared to 10–15 %) [5–7].

The Edinburgh Postnatal Depression Scale is a widely used 10-item self-report scale originally devised to screen for depression in the postnatal period but subsequently validated for antenatal use. There is a maximum score of 30 and women who score greater than 12 are generally considered to be likely to meet criteria for a formal diagnosis of clinical depression. Migrants, asylum seekers and refugees have been found to be significantly more likely to score greater than 10 on the EDS and have lower scores for social support [6]. Culturally some groups such as South-East Asian women avoid discussion of personal physical or mental problems outside their family due to fears of stigma [8, 9] and may manifest these problems in other ways, such as with somatic disorders [10].

In response to the increasing recognition that social and emotional problems in the perinatal period can lead to worse outcomes for women and their babies, a number of Australian jurisdictions have introduced psychosocial assessment including depression screening. This process was facilitated by *beyondblue, the national depression initiative*, in various ways, including production of perinatal clinical practice guidelines for primary care professionals [11]. In NSW, the Supporting Families Early Policy has integrated psychosocial risk assessment with routine care (Integrated Perinatal Care; IPC) during pregnancy and after the birth, providing a coordinated network of support and services for mothers and their babies [12, 13]. All women receive this assessment from midwives at the antenatal booking visit, then from the Child and Family Health Nurse (CFHN) at a universal home visit following birth and again at the 6–8 week check at the Early Childhood Centre. It includes the EPDS and a series of questions that reflect seven key variables or domains of risk (Tables 1 and 2). Concerns have been expressed about specific skills required in understanding, interpreting and responding appropriately to women’s needs and to the adequacy of midwives’ skills and the support provided to them [13, 14]. This becomes even more complex where migrant women are concerned when cultural understandings, taboos and language barriers come into play.

**Table 1:** Psychosocial risk variable s I–VII. NSW Department of Health (2009)

Variables (Risk factors)	Suggested format for psychosocial assessment questions
I. Lack of support	1. Will you be able to get practical support with your baby?
	2. Do you have someone you are able to talk to about your feelings or worries?
II. Recent major stressors in the last 12 months.	3. Have you had any major stressors, changes or losses recently (ie in the last 12 months) such as, financial problems, someone close to you dying, or any other serious worries?
III. Low self-esteem (includung lack of self-confidence, high anxiety and perfectionistic traits)	4. Generally, do you consider yourself a confident person?
	5. Does it worry you a lot if things get messy or out of place?
IV. History of anxiety, depression or other mental health problems	6a. Have you ever felt anxious, mesirable,worried or depressed for more than a couple of weeks?
	6b. If so, did it seriously interfere with your work and your relationships with friends and family?
	7. Are you currently receiving, or have you in the past received, treatment for any emotional problems?
V. Couple’s relationship problems or dysfunction (if applicable)	8. How would you describe your relationship with your partner?
	9. a). Antenatal: What do you think your relationship will be like after the birth
	OR
	9. b). Postnatal (in Community Health Setting): Has your relationship changed since having the baby?
VI. Adverse childhood experiences	10. Now that you are having a child of your own, you may think more about your own childhood and what it was like.
	As a child were you hurt or abused in any way (physically, emotionally, sexually)?
VII. Domestic violence.	11. Within the last year have you been hit, slapped, or hurt in other ways by your partner or ex-partner?
Questions must be asked only when the woman can be interviewed away from partner or family member over the age of three years. Staff must undergo training in screening for domestic violence before administering questions	12. Are you frightened of your partner or ex-partner? (If the response to questions 11 & 12 is “No” then offer the DV information card and omit questions 13–18)
	13. Are you safe here at home? /to go home when you leave here?
	14. Has your child/children been hurt or witnessed violence?
	15. Who is/are your children with now?
	16. Are they safe?
	17. Are you worried about your child/children’s safety?
	18. Would you like assistance with this?
Opportunity to disclose further	19. Are there any other issues or worries you would like to mention?

**Table 2:** Psychosocial questions as contained in ObstetriX™

History of mental health treatment	Text field
Pattern of alcohol consumption	Text field
Illegal drug usage	Text field
Drug support needed	Text field
Currently on drug support	Text field
“Is there someone to talk to about your feelings or worries?”	Yes / no / not sure / unable to ask
“Will you be able to get practical support after the birth of your baby?”	Yes / no / not sure / unable to ask
“In the last 12 months have you had any major worries, stress or change?”	no / financial difficulties / housing changes / relationship worries / significant isolation / loss or death / other / unable to ask
“Generally do you consider yourself a confident person?”	No / yes / sometimes / unable to ask
“Does it worry you a lot if things get messy or out of place?”	No / yes / sometimes / unable to ask
“Have you ever felt anxious or depressed for more than 2 weeks?”	No / yes / unable to ask
If yes, “did any episode seriously interfere with your work or relationships?”	No / yes
And “were any of these episodes of anxiety related to pregnancy or birth?”	No / yes PND / yes – postnatal psychosis / other / not known
Child living away	Text field
Department of Community Services	Text field
Frightened by partner	Text field
Would you like assistance with this	Text field
Response to DV questions	Text field
Other issues or worries	Text field
EDS total score	Text field
Answer to EDS question 10	Text field
Psychosocial assessment – issues identified?	Text field

As part of the introduction of routine and universal psychosocial assessment including the EPDS, it was necessary to ensure workforce education and support and also relevant referral resources. In NSW, inpatient mother-baby psychiatric unit beds are available only in the private health system at St John of God Hospital in Sydney, so it was vital that specialised services were made available to manage disorders requiring more than primary care. Following successful establishment of IPC and secondary level perinatal and infant mental health services (PIMH) in the public health system in Sydney’s South West, members of the team accepted an invitation from St John of God Health Care NSW, to establish a community PIMHS. This clinic, known as the Raphael Centre, is in  Blacktown in Sydney’s west. It is one of eight similar PIMHS in Australia, and aims to provide comprehensive secondary, specialised mental health care for women and their partners and families from conception to when the index child reaches the age of four years.

The aim of this study is to evaluate whether health outcomes, including psychosocial factors vary between Australian and non-Australian born women in the maternity setting and to explore the effect of psychosocial factors on pregnancy progression events and birth outcomes.

## Methods

### Setting

Blacktown Hospital, located in Western Sydney, New South Wales, Australia, provides one of the state’s largest maternity services, with approximately 3000 births per year and is classified as a Level 4 Maternity Unit. Western Sydney is a diverse and highly populated area, characterised by an expanding population (with a high proportion of young families), multiculturalism (57 % not born in Australia) and widespread socio-economic disadvantage [15]. Antenatal psychosocial assessment, including depression screening, was introduced at Blacktown Hospital in June 2011. The questions contained in the screening tool are based on a series of known risk factors and are administered parallel to the EPDS. This was undertaken by the booking midwife in the privacy of the booking visit. If a NSW Health Interpreter was booked for the visit, the questions were asked verbally via the interpreter.

### Data

This study was a retrospective review of routinely collected data for a consecutive cohort of women who delivered babies at Blacktown Hospital between July 2012 and June 2013. Data was sourced from the WSLHD ObstetriX™ database, an information system that collects clinical data from first antenatal visit, through to discharge of mother and baby from the hospital.

Variables of interest included (i) demographics, (ii) obstetric characteristics and medical risks, (iii) psychosocial risks, (iv) depressive and anxiety symptoms, (v) referrals to perinatal services, (vi) delivery details, (vii) postnatal outcomes and (viii) breastfeeding. The relationship between psychosocial risk and health outcomes were also examined.

### Analysis

Raw EPDS scores, collected by clinical staff at the first antenatal visit, were grouped to form the dichotomous variable EPDS ≤ 12 or EPDS ≥ 13 for all women. Women were grouped in non-Australian born and Australian born cohorts and for the non-Australian born cohort, the seven most commonly occurring countries of birth were examined independently. Pregnancy, labour and delivery events were then analysed utilising contingency tables and chi square results were calculated. Logistic regression techniques were applied and reported as unadjusted and adjusted odds ratios and 95 % confidence interval following adjustment for maternal age, gestation at birth, maternal body mass index, country of birth and smoking. Analysis was undertaken with IBM SPSS v.23™. Due to the number of statistical tests undertaken, a *p* value < 0.025 was set for significance.

Ethics approval was given by WSLHD Protocol Number HREC2013/4/6.7(3697) AU RED LNR/13/WMEAD/98. There was no need for individual consent to be obtained from the participants as we only received de-identified data and so could in no way identify participants. Western Sydney Local Health District Human Rights Ethics Committee approved this.

## Results

In total, there were 3092 women in the cohort. The average age of women in this sample at booking-in was 29.17 years (SD: 5.49, range: 15–47 years). More than half (56.7 %) were aged 20–35 years, 25.4 % were older than 35 years and 4 % were aged less than 20 years. The average gestation at booking-in was 17.18 weeks (SD: 6.12, range: 1–41 weeks), with the majority (62.9 %) booking in between 13 and 19 weeks. The rates of public, private and overseas bookings (i.e. with no access to universal health care) were 94.7 %, 3.1 % and 2.2 %, respectively. The percentage of women receiving their antenatal care by hospital-based midwives was 55.4 %, with 39.7 % of women cared for antenatally by hospital doctors and 3 % cared for by private obstetricians. General practitioner and ‘other’ antenatal care providers combined to care for the remaining 1.9 % of women.

The cohort was ethnically diverse, with 56.6 % having been born outside Australia. The most common countries of birth outside Australia were India (14.0 %), the Philippines (7.4 %), New Zealand (4.8 %), Fiji (3.4 %), Sudan (2.3 %), Pakistan (2.2 %) and China (2.2 %). Almost one in ten women (8.6 %) needed an interpreter for hospital maternity appointments. Women who were born in Australia (*n* = 1341), compared to those not born in Australia (*n* = 1751), were younger; more likely to be a teenager and less likely to be over 35 years of age. They were also more likely to be having their first baby, six times more likely to smoke, more likely to be overweight, more likely to have gestational hypertension (GH), more likely to have gestational diabetes (GDM), more likely to have a normal vaginal birth, have an epidural and have some analgesia during the first stage of labour. Their babies were also slightly heavier (Table 3).

**Table 3:** Selected demographics, maternal obstetric history and maternal and neonatal outcomes among Australian-born and non-Australian-born women, *N* = 3092

	Australian-born *n* = 1341	Non-Australian-born *n* = 1751	*p*
Maternal age*	28.0 (5.88)	30.0 (4.99)	<0.0001
Teenage pregnancy	7.8 %	1.5 %	<0.0001
Pregnancy ≥35 years	14.7 %	19.0 %	0.002
Nulliparous	28.6 %	27.5 %	0.52
Smoking	19.7 %	3.0 %	<0.0001
BMI ≥ 30	32.0 %	21.4 %	<0.0001
Gestational Diabetes	6.8 %	13.7 %	<0.0001
Instrumental delivery	9.6 %	12.0 %	0.04
Caesarean section	27.0 %	30.0 %	0.06
Epidural use	26.2 %	20.2 %	<0.0001
Episiotomy	18.0 %	15.5 %	0.06

*Mean, SD and t-test, Median, IQ range, Mann–Whitney U

When we looked at women from the top seven countries of birth compared to Australian born women we found other significant variations when compared to Australian born women. Women born in China had the highest rates of GDM (20.3 % v 6.8 %). The highest rates of GH were seen in The Philippines cohort (5.3 % v 4.4 %), where as higher rates of maternal anaemia were seen in women from Pakistan (15.9 % v 9.3 %).

Psychosocial risk factors also varied between Australian-born and non-Australian born women. Women born in Australia (and New Zealand) were much more likely to smoke and report a mental health disorder. Chinese women were more likely to report illegal drug use While not statistically significant, other differences noted were: higher rates of thoughts of self-harm and a EPDS score of ≥13 in women born in the Philippines, and more domestic violence, family history of mental illness and other disability reported by women born in Pakistan. Sudanese women reported less home and emotional support. It was interesting to note that women from several countries reported no mental health disorders (India, Fiji, Sudan and Pakistan) despite high reports, for example, of a family history of mental health disorder (Pakistan) and anxiety and depression risk (India) (Table 4).

**Table 4:** Country of birth and associated psychosocial risk factors

	Australia	India	Philippines	New Zealand	Fiji	Sudan	China	Pakistan	Other	*p*
	*n* = 1341	*n* = 433	*n* = 229	*n* = 147	*n* = 104	*n* = 71	*n* = 69	*n* = 69	*n* = 629	
Thoughts of self harm	1.1 %	0.5 %	**2.5 %**	1.6 %	0.0 %	0.0 %	0.0 %	0.0 %	2.0 %	0.23
EPDS ≥ 13	4.3 %	5.9 %	**9.0 %**	3.9 %	3.6 %	3.6 %	5.4 %	6.7 %	5.2 %	0.30
Domestic violence any	2.5 %	2.5 %	3.1 %	1.4 %	2.9 %	1.4 %	2.9 %	**4.3 %**	3.0 %	0.95
Frightened by partner	0.8 %	0.7 %	0.4 %	0.0 %	0.0 %	0.0 %	0.0 %	**1.4 %**	1.0 %	0.56
Smoking risk	**19.7 %**	0.0 %	0.9 %	**19.7 %**	1.9 %	1.4 %	0.0 %	0.0 %	3.0 %	<0.001
Illegal drug use risk	0.5 %	0.2 %	0.4 %	0.0 %	1.0 %	0.0 %	**2.9 %**	0.0 %	1.6 %	0.04
Alcohol consumption risk	0.3 %	0.2 %	0.4 %	**1.4 %**	1.0 %	0.0 %	0.0 %	0.0 %	1.1 %	0.28
Childhood abuse	8.4 %	8.4 %	8.1 %	**10.4 %**	10.3 %	6.7 %	6.6 %	7.9 %	11.2 %	0.66
Pregnancy-related anxiety risk	2.0 %	2.0 %	1.4 %	2.2 %	2.1 %	3.3 %	**4.8 %**	1.6 %	2.3 %	0.91
Work/relationship effect risk	8.2 %	7.9 %	4.7 %	8.1 %	5.2 %	**11.7 %**	4.8 %	6.3 %	10.0 %	0.32
Anxiety/depression risk	13.5 %	**16.8 %**	12.8 %	15.6 %	8.2 %	15.0 %	11.3 %	9.4 %	16.1 %	0.32
Worried about mess risk	9.1 %	12.5 %	**12.8 %**	10.4 %	6.2 %	11.7 %	9.7 %	9.4 %	13.8 %	0.10
Generally confident risk	2.8 %	2.3 %	4.7 %	0.7 %	2.1 %	1.7 %	**4.8 %**	1.6 %	3.3 %	0.47
Recent worry/stress risk	21.9 %	23.6 %	30.3 %	23.5 %	24.7 %	20.0 %	**30.6 %**	23.4 %	26.7 %	0.15
Home support risk	5.5 %	4.8 %	4.8 %	3.4 %	5.8 %	**9.9 %**	5.8 %	4.3 %	5.2 %	0.80
Emotional support risk	3.3 %	3.3 %	2.4 %	2.2 %	5.2 %	**6.7 %**	3.2 %	0.0 %	4.7 %	0.34
Mental health treatment risk	**18.8 %**	2.3 %	4.4 %	7.5 %	5.8 %	5.6 %	2.9 %	4.3 %	7.8 %	<0.001
Mental health disorder	**3.1 %**	0.0 %	1.3 %	1.4 %	0.0 %	0.0 %	1.4 %	0.0 %	0.6 %	<0.001
Family history mental health disorder	11.3 %	11.2 %	10.8 %	11.8 %	9.3 %	8.2 %	4.8 %	**17.2 %**	13.6 %	0.39
Mental health vulnerability risk	**37.7 %**	28.6 %	29.3 %	33.3 %	28.8 %	29.6 %	24.6 %	31.9 %	31.6 %	<0.001
Other disability	6.3 %	6.0 %	7.0 %	1.4 %	8.7 %	4.2 %	4.3 %	**10.1 %**	7.9 %	0.14

Bolded % indicate highest reportes levels

There was an association with having a EPDS ≥ 13 and lower breastfeeding rates at discharge (Table 5) and also with other psychosocial issues such as thoughts of self-harm, domestic violence, childhood abuse, anxiety and depression, lack of confidence, worry about mess, recent worry/stress and emotional support (Table 6). We examined women with an EPDS ≥ 13 at booking and the incidence of pregnancy conditions and events compared to women with EPDS ≤12 adjusting for smoking, parity, age, Body Mass Index (BMI), and Australian or non-Australian born, and found significant associations with a higher EPDS and GDM (AOR 1.85 95 % CI 1.14–3.0) and antepartum haemorrhage (APH) (AOR 2.69 95 % CI 1.02–7.00 (Table 7). Only the association between EPDS scores and GDM reached the pre-determined *p* < 0.025.

**Table 5:** Demographic and pregnancy details between women with an EDS ≤ 12 at booking and those with an EPDS ≥ 13

	EPDS ≤12	EPDS of ≥13	*p*
Age	29.1 (5.47)	29.8 (5.78)	0.16
Primiparous	28.8 %	25.9 %	0.48
Born in Australia	43.9 %	37.0 %	0.12
BMI ≥ 30	26.9 (6.61)	25.8 (6.81)	0.06
Smoking	10.3 %	11.9 %	0.56
Assisted Reproductive Technology	2.9 %	1.5 %	0.33
Vaginal delivery	72.0 %	71.8 %	0.97
Apgar 5 minutes	9 (8–9)	9 (8–9)	0.16
Birth weight	3354 (599.4)	3305 (664.7)	0.36
Breast Feeding at discharge	79.0 %	69.1 %	0.003

**Table 6:** Associated psychosocial issues for women with an EDS ≤ 12 at booking compared to those with an EPDS ≥13

	EPDS ≤12	EPDS of ≥13	*p*	OR
Mental health vulnerability risk	55.4 %	100 %	<0.001	1.8 (1.74–1.87)
Thoughts of self harm	0.5 %	15.6 %	<0.001	38.35 (18.4–79.87)
Domestic violence – any	2.2 %	14.1 %	<0.001	7.31 (4.20–12.72)
Smoking risk	10.3 %	11.9 %	0.56	1.17 (0.69–2.01)
Illegal drug use risk	0.6 %	0.0 %	0.35	0.99 (0.99–1.00)
Alcohol consumption risk	0.5 %	0.0 %	0.40	1.00 (0.99–1.00)
Childhood abuse	8.4 %	18.5 %	<0.001	2.49 (1.58–3.93)
Pregnancy related anxiety risk	1.6 %	11.9 %	<0.001	8.52 (4.63–15.68)
Work/relationship effect risk	6.6 %	36.3 %	<0.001	8.10 (5.51–11.90)
Anxiety/depression risk	12.2 %	51.1 %	<0.001	7.56 (5.28–10.81)
Worried about mess risk	9.5 %	37.0 %	<0.001	5.62 (3.86–8.16)
Positive response to ‘are you generally confident’ question	97.8 %	85.2 %	<0.001	0.13 (0.07–0.22)
Recent worry/stress risk	22.1 %	63.7 %	<0.001	6.20 (4.31–8.91)
Emotional support risk	3.1 %	10.4 %	<0.001	3.61 (1.99–6.56)
Mental health disorder	1.7 %	0.7 %	0.39	0.43 (0.06–3.13)
Family history of mental health disorder	11.6 %	11.1 %	0.85	0.95(0.55–1.65)

**Table 7:** Odds ratio calculations for women with an EPDS ≥ 13 at booking and pregnancy conditions and events when compared to women with an EPDS ≤12 (ref category is EPDS < 13)

	OR	AOR	*p*
Gestational Diabetes	1.75 (1.09–2.82)	1.85 (1.14–3.03)	0.01
Assisted Reproductive Technology	0.50 (0.12–2.07)		
Ante Partum Haemorrhage	3.39 (1.40–8.23)	2.69 (1.02–7.00)	0.05
Caesarean section	1.08 (0.70–1.66)		
Instrumental delivery	0.94 (0.50–1.78)		
Birth <37 weeks*	1.43 (0.56–3.64)		
Episiotomy	1.16 (0.74–1.81)		
Epidural use	0.95 (0.60–1.51)		
Smoking	1.17 (0.69–2.00)		
Previous pregnancy	1.15 (0.78–1.71)		

*non-tertiary hospital

All significant ORs adjusted for smoking (yes/no), primip (yes/no), age (<20, 20–34, ≥35), BMI (obese yes/no), born in Australia (yes/no)

## Discussion

In this study of one hospital with a large number of migrant women giving birth in Western Sydney we found differences in demographics and obstetric outcomes that we have identified previously in the state-wide population [3]. Women born in Australia tend to be younger when they give birth compared to those women born overseas and their babies are slightly heavier.

When looking at the top seven countries from which women giving birth in this unit came, other country specific differences could be seen. Once again the high rate of GDM was seen amongst women born in China and a high caesarean section rate was seen for women born in India. Indian women also had a much higher rate of private health insurance and previous work we have undertaken has demonstrated this and the link between this and high obstetric intervention rates [16, 17].

This study looked at the variations in psychosocial risk factors among women from different countries, showing that there continues to be a need to tailor care more specifically to different migrant groups. For example, the fact that women from several countries reported no mental health disorders despite some of them reporting high rates of a family history of mental health disorders (eg Pakistan) and anxiety and depression (India) is concerning. Issues include: how questions are posed, whether they are culturally acceptable or able to be interpreted correctly, and who is present at the consultation. Concerns have previously been expressed about the integration of psychosocial assessment into routine clinical care [13], especially from non-English speaking backgrounds [18]. Other researchers have argued that the assessment of social and emotional health needs require specific skills in understanding interpreting and responding to women’s needs [19] and this is even more complex when dealing with women from a migrant background. A study observing midwives undertaking psychosocial screening found that while many midwives demonstrated skills in undertaking psychosocial assessment and responded appropriately, there were also many instances where this could have been improved [13]. Provision of appropriate pathways to care is equally vital.

This is the first time to our knowledge that an association has been found between an EPDS ≥13 at booking and the increased incidence of GDM. The fact that the EPDS is done before the screening test for GDM makes this particularly interesting as this is potentially a causal association. A bidirectional diabetes-depression relationship has been found in women aged 50–75 when adjustments are made for diabetes and related co-morbidities [20]. In a study of 55,000 US women over 10 years researchers found depression and diabetes were closely related to each other and this reciprocal association depended on the severity or treatment of each condition [20]. While previous studies have shown the odds of depression is about twice that in diabetic groups compared to non-diabetic groups, this has been explained as a consequence of having diabetes. In the Pan et al. study the authors found depression had an effect on the incidence of diabetes independent of adiposity and inactivity and those treated with antidepressant medication were at even higher risk of developing type 2 diabetes. Whether this is due to the fact that antidepressant use reflects the severity of the depression or antidepressants exert some clinical effects on glucose homeostasis is still debated. The underlying mechanism for this link needs to be established [20]. A similar bidirectional association has been found between depression and Metabolic Syndrome (central obesity, hyperglycemaia, elevated blood pressure, hypertriglyceridemia, decreased HDL Cholesterol) [21] and depression and insulin resistance [22].

The pathophysiology of this link could be due to hypothalamic pituitary adrenal axis hyperactivity (HPA) and mental stress induced symphathomedullary activation in patients with major depression leading to decreased glucose transport and insulin resistance. Cortisol and catecholamines are also increased with mental stress and depression causes inactivity, which combine with increased cortisol levels, increased adiposity and insulin resistance [23].

A link between a history of depression and GDM has been found in a multiethnic US cohort study [24] and there is some evidence that treating GDM reduces the risk for postpartum depression [25]. This is the first study we know of that shows an association between a booking EDS of ≥13 and the onset of GDM in the pregnancy.

Previous associations have been found between antepartum depressive symptoms and adverse obstetric and neonatal outcomes, such as increased admission of the baby to NICU [26]. Untreated depression impacts on nutrition, and poor compliance with care recommendations as well as increased alcohol and substance use, which all impact on perinatal outcomes [26]. Increased uterine artery resistance has been shown to be associated with maternal anxiety during pregnancy [27] along with low birth weight and preterm birth [28]. To our knowledge this is the first time an EPDS ≥13 on booking has been found to be associated with an increased risk of antepartum haemorrhage specifically, though using a pre-determined *p* < 0.025 this was not significant. Larger studies are needed to determine if there is an indeed and association.

There are several limitations with this study and these include that it involves only one hospital in the West of Sydney over a year. A larger sample size is needed to strengthen the association between a EPDS of ≥13 and the development of GDM as conducting numerous statistical tests on a smaller sample size does increase the risk of a Type I error. Also the division of non-Australian born women into countries also dilutes the data pool and limits conclusions about individual groups. The advantages of using the ObstetriX™ database are the large number of variables available compared to the other state-wide routine data bases, such as the Perinatal Data Collection (PDC) and Admitted Patient Data Collection (APDC). Socioeconomic factors which affect health such as body mass index, psychosocial risk factors, marital status, education level, occupation, are not collected in the latter and adjustment for these variables cannot be undertaken when modelling statistical interactions with these databases.

## Conclusion

There are significant differences in obstetric and psychosocial risk profiles and maternal and neonatal outcomes between Australian-born and non-Australian born women. For example, there appears to be a link between an EPDS of ≥13 and GDM which warrants further investigation. This and other results from the initial analysis will be investigated in a larger ObstetriX™ dataset.

## Abbreviations

GH: Gestational hypertension
GDM: Gestational diabetes mellitus
EDPS: Edinburg postnatal depression score
APH: Antepartum haemorrhage
SCN: Special care nursery
NICU: Neonatal intensive care unit
DSM-V: Diagnostic and statistical manual of mental disorders fifth edition
PND: Postnatal depression
NSW: New South Wales
CFHN: Child and family health nurse
PIMHS: Perinatal and infant mental health services
IPC: Integrated perinatal care
WSLHD: Western Sydney local health district
BMI: Body mass index
APDC: Admitted patient data collection
PDC: Perinatal data collection
